# Sucrose metabolism in developing oil-rich tubers of *Cyperus esculentus*: comparative transcriptome analysis

**DOI:** 10.1186/s12870-018-1363-9

**Published:** 2018-07-24

**Authors:** Zhenle Yang, Dantong Liu, Hongying Ji

**Affiliations:** 1 Key Lab of Plant Resources, Institute of Botany, Chinese Academy of Sciences, Beijing, 100093 China; 2 University of Chinese Academy of Sciences, Beijing, 100049 China

**Keywords:** *Cyperus esculentus*, Oil-rich tuber, Sucrose metabolism, Enzymatic pathways, Gene expression pattern

## Abstract

**Background:**

*Cyperus esculentus* is unique in that it can accumulate significant amounts of oil, starch and sugar as major storage reserves in tubers with high tuber yield and therefore considered as a novel model to study carbon allocation into different storage reserves in underground sink tissues such as tubers and roots. Sucrose (Suc) plays a central role in control of carbon flux toward biosynthesis of different storage reserves; however, it remains unclear for the molecular mechanism underlying Suc metabolism in underground oil-rich storage tissues. In the present study, a comprehensive transcriptome analysis of *C. esculentus* oil tuber compared to other plant oil- or carbohydrate-rich storage tissues was made for the expression patterns of genes related to the Suc metabolism.

**Results:**

The results revealed some species-specific features of gene transcripts in oil tuber of *C. esculentus*, indicating that: (i) the expressions of genes responsible for Suc metabolism are developmentally regulated and displayed a pattern dissimilar to other plant storage tissues; (ii) both of Suc breakdown and biosynthesis processes might be the major pathways associated with Suc metabolism; (iii) it was probably that Suc degradation could be primarily through the action of Suc synthase (SUS) other than invertase (INV) during tuber development. The orthologs of SUS1, SUS3 and SUS4 are the main SUS isoforms catalyzing Suc breakdown while the vacuolar INV (VIN) is the leading determinant controlling sugar composition; (iv) cytosolic hexose phosphorylation possibly relies more on fructose as substrate and uridine diphosphate glucose pyrophosphorylase (UGP) plays an important role in this pathway; (v) it is Suc-phosphate synthase (SPS) B- and C-family members rather than SPS A that are the principal contributors to SPS enzymes and play crucial roles in Suc biosynthesis pathway.

**Conclusions:**

We have successfully identified the Suc metabolic pathways in *C. esculentus* tubers, highlighting several conserved and distinct expressions that might contribute to sugar accumulation in this unique underground storage tissue. The specific and differential expression genes revealed in this study might indicate the special molecular mechanism and transcriptional regulation of Suc metabolism occurred in oil tubers of *C. esculentus*.

**Electronic supplementary material:**

The online version of this article (10.1186/s12870-018-1363-9) contains supplementary material, which is available to authorized users.

## Background

*Cyperus esculentus* (commonly known as yellow nutsedge or tiger nut) is a grass-like perennial herb that belongs to the sedge family Cyperaceae that is widely distributed in almost all tropical, subtropical and temperate zones [1]. *C. esculentus* is currently found a special untraditional underground crop since it has relatively high yield (ranging from 4.5 to 12 t·ha^− 1^) [2] and its storage tubers accumulate high levels of all three storage reserves: up to 40% starch, 35% oil, and 20% soluble sugars [3–6], which offers it high energy with ~ 400–450 kcal/100 g [6]. Tubers of *C. esculentus* are edible and consumed in fresh, dried and roasted forms, or milled into flavor gluten-free flour or ground to make a delicious milk-like drink [6, 7]. The tubers can be used to produce edible oil which is comparable to olive or hazelnut oils [8, 9], and the defatted residue can be further applied for feedstuff or making sugar and brewing wine [10]. In addition, *C. esculentus* is of great benefit to human health [11, 12], and could be utilized as a potential good feedstock for biofuel production due to the high contents of oil and starch [13–15].

The accumulation patterns of major storage reserves in developing tubers of *C. esculentus* were previously characterized by biochemical analysis, which indicated that oil, starch and sugars were accumulated in an orderly and gradually way in the developing tubers [4]. Therefore, *C. esculentus* was regarded as a novel model plant to study carbon partitioning into different storage reserves in non-photosynthetic underground storage tissue. However, the molecular mechanism underlying carbon flux allocation of *C. esculentus* tuber remains unclear.

As an important carbon metabolic process, Suc metabolism is not only crucial for plant growth and development by providing with carbon source and energy [16, 17], but also a determinant for sugar accumulation and pivotal to Suc homeostasis conferring tolerance to biotic and abiotic stresses [18–21]. In particular, Suc metabolism plays a central role in control of carbon flux toward biosynthesis of different storage reserves including starch, oil, sugar, and protein in sink tissues such as seeds, fruits, tubers, stems, bulbs, meristems and flowers [16].

Despite its importance, a description of the comprehensive processes or pathways of Suc metabolism in underground oil-rich storage vegetative tissues is still missing. Although Suc metabolism in model plants is well documented and its process is considered to be a highly conserved among plants, the molecular and biochemical details in oil-rich tuber plants remain elusive. Moreover, the molecular regulatory mechanism underlying Suc metabolism in oil tubers other than oil-rich seeds and fruits, or carbohydrate-rich fruits, roots and tubers is still largely unknown. In this study, we made a comprehensive analysis of the global gene expressions related to Suc metabolism in developing tubers of *C. esculentus* by comparison with other plant oil- or carbohydrate-rich storage organs, and revealed several species-specific gene expression profiles. Our study represents a comprehensive analysis of the genes involved in Suc metabolism in *C. esculentus*, which will not only shed light on the Suc metabolism in oil tuber tissues, but also provide a foundation for further research on the functions of these genes and subsequent manipulation of Suc metabolism with the aim to increase the accumulation of oil, starch or sugar, as well as the genetic improvement of the quality and yield of *C. esculentus* tuber.

## Results

### Sucrose accumulation in developing tubers of *C. esculentus*

Compared to other common plants that rich in oil or starch or sugars in their sink organs, the portion of sugars on fresh weight (FW) in mature tubers of *C. esculentus* is relatively high among the storage reserves, in which it is higher than those of oil plants and even greater than some carbohydrate-rich fruit, root and tuber crops such as grape, sugar beet, sweet potato and potato (Table 1). In tubers of *C. esculentus*, the major component of sugars is Suc that can accumulate up to 20% of dry weight, while the minor ones include glucose (Glc) and fructose (Fru) [4, 6, 22].

**Table 1:** Proximate composition (% of fresh weight) of plant storage tissues rich in oil or carbohydrate

Plant storage tissues	Plant	Starch	Lipid	Sugars	Protein	Fiber	Moisture	Reference
Seed	*Arabidopsis thaliana* (Ws)	0.03	36.0	2.1	30.0		7.0	[23]
	Rape seed (*Brassica napus*)	0.6	44.3	16.6	10.8		5.0	[90]
	Castor bean (*Ricinus communis*)	23.32	35.4	trace	28.63	1.68	7.43	[91]
	Soybean (*Glycine max*)	13.53	19.94	7.33	36.49	9.3	8.54	[92]
	Rice (*Oryza sativa*)	78.53	0.66	0.12	7.13	1.3	11.62	[92]
	Maize (*Zea mays*)	10.44	1.35	6.26	3.27	2.0	76.05	[92]
Fruit	Avocado (*Persea americana*)	1.54	15.41	0.3	1.96	6.8	72.33	[92]
	Oil palm (*Elaeis guineensis*)	9.8	81.9	1.0	2.2	3.8	trace	[92]
	Date palm (*Phoenix dactylifera*)	1.8	0.15	66.47	1.81	6.7	21.32	[92]
	Grape (*Vitis vinifera*)	trace	0.1	6.98	0.63	1.1	90.89	[92]
Root	Sugar beet (*Beta vulgaris*)	trace	0.17	6.76	1.61	2.8	87.58	[92]
	Sweet Potato (*Ipomoea batatas*)	10.29	3.88	6.63	0.66	0.88	70.54	[93]
Tuber	Potato (*Solanum tuberosum*)	13.82	0.16	0.47	1.97	0.68	78.75	[94]
	Tigernut (*Cyperus esculentus*)	29.90	24.49	15.42	5.04	8.91	26.00	[95]

**Fig. 1:**
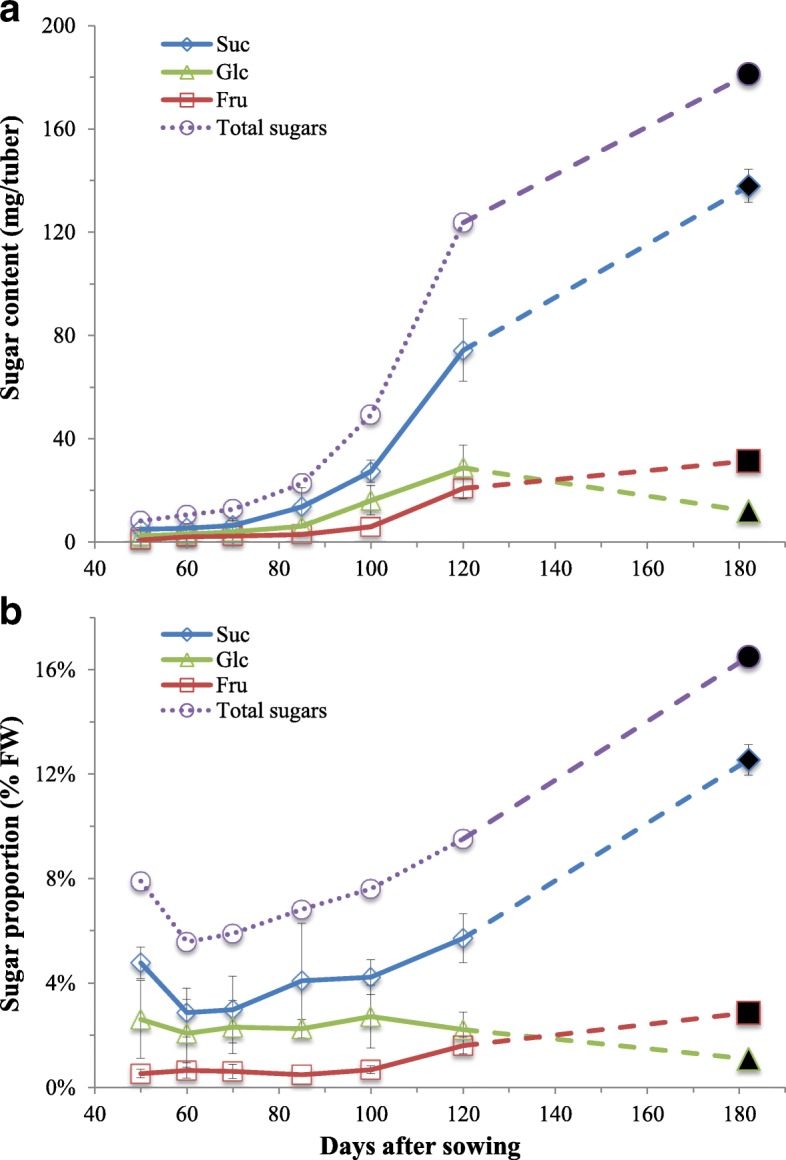
Percentage of sugars in fresh nutsedge tubers during tuber development. Values represent means±SD (*N* = 5). Black solid markers on the right indicate levels of sugars in seed tubers stored for 6 months at 4°C. **a** and **b**: Suc, sucrose; Glc, glucose; Fru, fructose. The levels of totalsugars were calculated as the sum of Suc, Glc, and Fru

To examine the effects of tuber developmental process on sugar accumulation, the sugar contents in developing tubers were determined in this study. The levels of total sugars were calculated as the sum of Suc, Glc, and Fru. The variation of sugar accumulation over the course of nutsedge development spanning around 120 days after seed tuber sowing (DAS) was shown in Fig. 1, which clearly indicated that there were different contents of individual and total sugars at different developmental stages. In all stages of development Suc concentration was highest followed by Glc with Fru being the least abundant. On a per fresh tuber basis, sugar accumulation in tubers was continuous throughout development, where sugar content ranged from 9 to 124 mg⋅tuber^− 1^. There was relatively slow sugar accumulation during early- to mid-stages (40–85 DAS), where the accumulation rate was only 0.41 mg⋅tuber^− 1^⋅d^− 1^, much lower than that of late stage (85–120 DAS) with 2.88 mg⋅tuber^− 1^⋅d^− 1^, suggesting that there existed differences in the metabolic processes regulating sugar accumulation in developing tubers. At 120 DAS, sugar was increased to 124 mg·tuber^− 1^, where Suc became much higher than Glc and Fru (Fig. 1a). At this point, sugar represented about 9.5% of FW, with Suc, Glc and Fru being 5.7, 2.2 and 1.6%, respectively (Fig. 1b). Suc turnover could theoretically generate more Fru over Glc via both INV and SUS catalysis. However, our results showed that Fru remained at lower levels than Glc throughout the tuber development. It was possibly that a higher demand of Fru substrate rather than Glc for hexose metabolism might occur in nutsedge tuber.

The patterns of Suc accumulation in *C. esculentus* during tuber development were similar to those occurred in developing sink organs for seeds of Arabidopsis [23, 24], rapeseed [25], tobacco [26], soybean [27] and fruits of tomato [28], melon [29], apple [30], pineapple [31], peach [32], jackfruit [33], and swelling tuber of potato [34], though the actual Suc contents during Suc accumulation were varying among the different plants. Temporal changes in Suc levels during tuber development clearly indicated that Suc accumulation in *C. esculentus* is highly regulated by developmental processes, which is likely associated with the expression patterns of Suc metabolism genes.

It was previously demonstrated that Suc accumulation was largely determined by the balance between Suc degradation (INV and SUS activities) and synthesis (SPS activity) [29]. A negative correlation has been observed between Suc accumulation and the activities of INV and SUS [35–37]. Although there remained controversial about the significant correlation with Suc accumulation in the case of SPS activity [35, 36], SPS seemed to play a pivotal role in regulation of Suc mobilization in the cycle of simultaneous Suc degradation and re-synthesis and therefore provide Suc utilization for metabolism [38, 39]. It was suggested that [4], when developing tuber of *C. esculentus* were transformed from cell-division into storage compartments where oil accumulation was commenced, there was a switch of main enzyme activity from INV to SUS-mediated Suc metabolism, leading to a shift from high hexose-to-Suc to high Suc-to-hexose in tubers. In this study, we indeed observed the high Suc-to-hexose ratios over the tuber development of *C. esculentus* (Fig. 1), where oil accumulation began at around 50 DAS [40].

Overall, our results revealed that the changes in sugar accumulation were associated with the tuber development.

### Sucrose metabolism-related genes in *C. esculentus* tubers display different transcriptional patterns from those of other plant oil-rich or carbohydrate-rich storage tissues

**Fig. 2:**
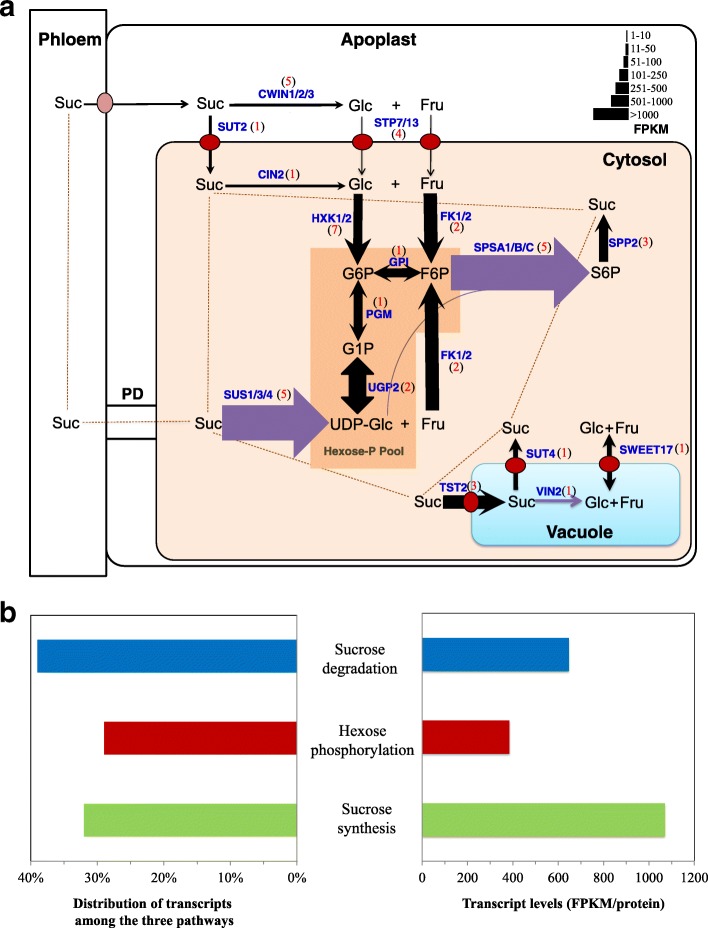
Gene expression pattern for Suc metabolism. **a** Schematic Suc metabolic pathways in oil-rich tubers of *Cyperus esculentus*. Enzyme or protein names are indicated in blue. For each enzyme or protein, the width of the arrow indicates the gene expression level (FPKM, average fragments per kilobase of exon model per million mapped reads). Black and purple arrows indicate respectively the common and different gene expression patterns in *Cyperus esculentus* tuber from those in other plant examined in this study. Transporter was marked by the black arrow across red oval. FPKM values of the different isoforms and/or subunits are average transcript levels. Red numbers in black bracket show the numbers of unigenes with more than 1 of FPKM for each enzyme or protein. Abbreviation: CIN, cytoplasmic invertase; CWIN, cell wall invertase; F6P, fructose-6-phosphate; FK, fructokinase; Fru, fructose; G1P, glucose-1-phosphate; G6P, glucose-6-phosphate; Glc, glucose; Hexose-P, hexose-phosphate; HT, hexose transporter; HXK, hexokinase; PD, plasmodesmata; PGI, phosphoglucoisomerase; PGM, phosphoglucomutase; S6P, succose-6-phosphate; SPP, sucrose-phosphatase.; SPS, sucrose-phosphate synthase; Suc, sucrose; SUS, sucrose synthase; SUT, sucrose transporter; T6P, trehalose-6-phosphate; TPS, trehalose-6-phosphate synthase; TST, tonoplast sucrose transporter; SWEET, Sugars Will Eventually be Exported Transporter (hexose and sucrose transporter); UDP-Glc, uridine diphosphate glucose; UGP, UDP-Glc pyrophosphorylase; VIN, vacuolar invertase. **b** Expression levels for Suc metabolism genes. Left, The distribution of transcript contents among the three pathways. Right, FPKM per protein in each pathway. Without regard to separation of close paralogs or allelic transcripts, multiple protein isoforms or subunits of a multi-protein complex were considered as a single protein and their transcripts were summed (Additional file 1: Table S3)

Our previous transcriptome analyses of *C. esculentus* tubers from three representative samples at 50, 85 and 120 DAS of developmental stages (i.e. the early stage of 40–50 DAS, the middle stage of 50–85 DAS and the late stage of 85–120 DAS, corresponding to the stage of low oil level and oil accumulation rate, medium oil level but high oil accumulation rate, and high oil level with low oil accumulation rate, respectively) have indicated that 41.05% of total 99,558 transcripts were successfully annotated [40]. Among them, 488 annotated transcripts were involved in carbohydrate metabolism, of which 355 unigenes were found to be expressed in the developing *C. esculentus* tubers (Additional file 1: Table S1d). More than 120 transcripts and 60 unigenes were identified for Suc metabolism (Additional file 1: Table S3). The majority of the Suc metabolism-related transcripts and unigenes were involved in hexose phosphorylation, followed by Suc degradation and Suc synthesis in this order (Fig. 2b, Additional file 1: Table S1d). Most of already known Suc metabolism enzymes and/or proteins were successfully detectable in tubers of *C. esculentus*, indicating their ability to participate in Suc metabolism in oil tubers, as similar to other plants.

Based on the gene expression of our transcriptome data and current model of plant Suc metabolism, the core Suc metabolic ways in *C. esculentus* tuber were illustrated in Fig. 2a. Suc metabolism primarily comprises of three metabolic pathways, i.e., Suc breakdown, phosphorylation of hexoses including Glc and Fru, and biosynthetic reactions of Suc from phosphorylated hexoses. Suc breakdown in the non-photosynthetic sink tissues involves two ways, hydrolysis and cleavage by various enzymes. Suc unloaded from the phloem may enter either apoplasmically into cell wall matrix or taken up intact symplasmically through plasmodesmata (PD) [41, 42], thus Suc can be either hydrolyzed by cell wall invertases (CWINs) into of its constituent monosaccharides such as Glc and Fru, or degraded by SUS into UDP-Glc and Fru, or by intracellular invertases such as cytoplasm invertases (CINs) and VINs, producing Glc and Fru [17]. Hexose phosphorylation mainly involves multiple pathways in which the resulting Glc, Fru and UDP-Glc are phosphorylated to glucose-6-phsophate (G6P), fructose-6-phosphate (F6P) and glucose-1-phsophate (G1P) by hexokinase (HXK), fructose-specific fructokinase (FK) and UGP, respectively. The reversible conversion of G6P into F6P or G1P is catalyzed by phosphoglucoisomerase (PGI) or phosphoglucomutase (PGM). Suc re-synthesis primarily involves two-step irreversible reactions where F6P combined with UDP-Glc is converted into Suc-6-phosphate (S6P) through the action of SPS, and subsequently S6P is converted into Suc and phosphate catalyzed by S6P phosphatase (SPP) [43].

In *C. esculentus,* the expressions of genes related to Suc synthesis among these metabolic pathways were most abundant, with an average value of transcript levels, represented by FPKM (fragments per kilobase of exon model per million mapped reads) per protein, almost equaling the total levels of Suc degradation and hexose phosphorylation (Fig. 2b). This result suggested that transcriptional regulation of Suc synthesis might be a major factor associated with Suc metabolism in *C. esculentus* tuber.

**Fig. 3:**
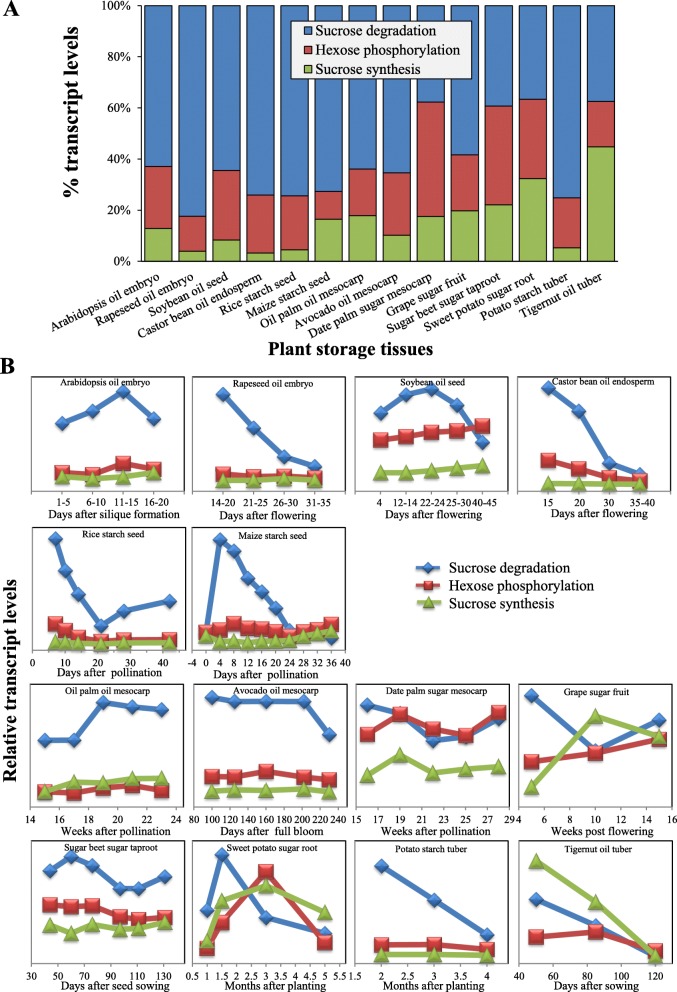
Expression patterns for three metabolic pathways of Suc metabolism in diverse plant storage tissues. **a** The relative distribution of transcripts among the selected three pathways related to Suc metabolism in diverse plant storage tissues. The data are averaged on all developing stages of plant tissues. **b** Temporal transcriptional levels during plant development. Transcriptome data of diverse plants are based on: (1) seeds of Arabidopsis [85], rapeseed [86], soybean [68], castor bean [86], rice [69], and maize [70]; (2) fruits of oil and date palm ([87], avocado [88], and grape [89]; (3) roots of sugar beet [67] and sweet potato [57]; and (4) tubers of potato [56] and tigernut (this study)

**Fig. 4:**
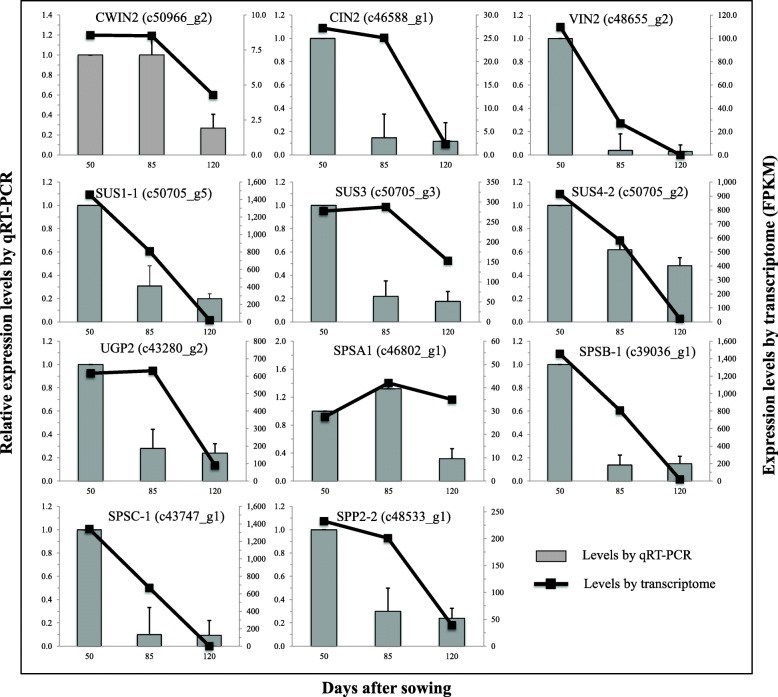
Relative gene expression profiles of select unigenes involved in Suc metabolism measured using qRT-PCR. 18 s rRNA was used as internal control for qRT-PCR. Values represent means±SD (*N = 4*). The primer sequences for these genes are listed in Additional file 1: Table S4

In addition, genes expressed in the three metabolic processes were all down-regulated across the tuber development, with different transcript levels at various stages of development (Fig. 3b), suggesting that genes associated with Suc metabolism in *C. esculentus* tuber are developmentally regulated. This is further confirmed by the results of quantitative realtime PCR (qRT-PCR) analysis for selected candidate genes associated with Suc metabolism in developing tubers (Fig. 4).

We found that, the Suc metabolism in *C. esculentus* tubers had different transcriptional patterns from those of other plant storage tissues such as oil-rich or carbohydrate-rich seeds, fruits, roots and tubers (Fig. 3). In contrast to *C. esculentus* tuber, the most highly expressed genes in other plant species were involved in Suc degradation, rather than Suc synthesis. Furthermore, the transcripts for Suc synthesis were much lower, with a 2- to 30-fold less than the total levels of Suc degradation and hexose phosphorylation (Fig. 3a). In addition, the temporal expression patterns of Suc metabolism were also dissimilar between *C. esculentus* tuber and other plant storage tissues during the course of development (Fig. 3b). In *C. esculentus* tuber, transcripts for the three Suc metabolic pathways were all down-regulated, and in particular, both of Suc degradation and synthesis displayed coordinated temporal expression during tuber development. In contrast, Suc synthesis genes in other plant storage tissues were expressed constantly or slightly up and showed a different pattern from those of Suc degradation across all the developmental stages.

Together, our data indicated that the genes related to Suc metabolic pathways in *C. esculentus* tubers have distinct transcriptional patterns from those of other plant oil-rich or carbohydrate-rich storage tissues, indicating a different regulation of gene expression present in *C. esculentus* tubers.

### Sucrose synthase transcripts are highly expressed during sucrose degradation in *C. esculentus* tubers

Before it is channeled into various pathways in different subcellular compartments, Suc must be either hydrolyzed irreversibly by INV into Glc and Fru, or converted reversibly but preferably cleaved with UDP into UDP-Glc and Fru catalyzed by SUS [44] (Fig. 2a).

**Fig. 5:**
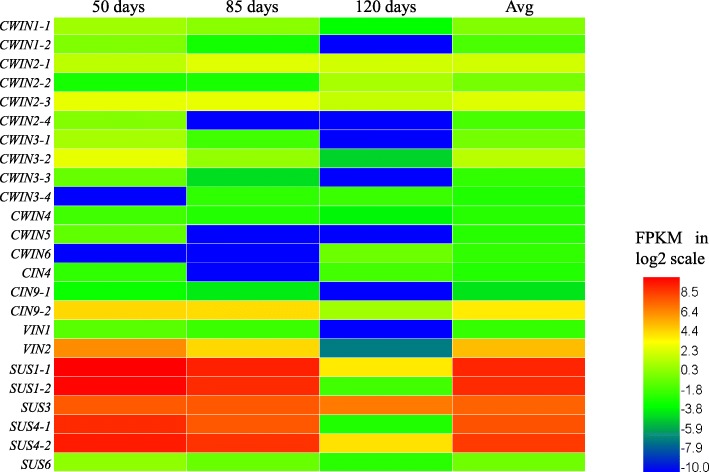
Expression profiles of Suc degradation genes at different tuber developmental stages. The values of expression levels (FPKM) were normalized with log2 using the HemI software

**Fig. 6:**
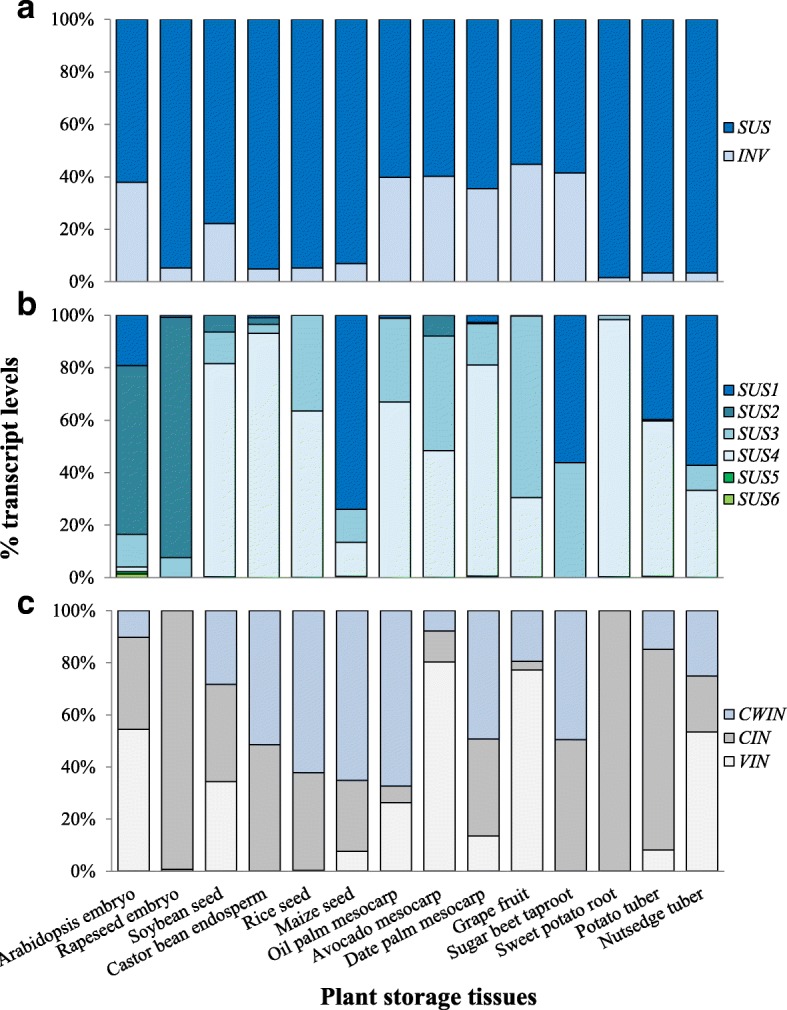
Expression patterns of Suc degradation genes in diverse plant storage tissues. **a** The relative distribution of transcripts of *SUS* over *INV*. **b** The relative distribution of transcripts of *SUS* isoforms. **c** The relative distribution of transcripts of *INV* isoforms

In *C. esculentus* tuber, the transcripts for the *SUS* genes in the cytosol were highly expressed, having a transcript value of more than 2500 FPKM/protein on average (Additional file 1: Table S3). The abundant expression levels of *SUS*, which were at least 10-fold higher than *INV*, also occurred across all the tuber development (Fig. 5). Much higher expression of *SUS* over *INV* in oil tuber is similar to other plant storage organs (Fig. 6a), indicating a conserved function of *SUS* in Suc metabolism among diverse plant storage tissues. In addition, our data showed that the change trend in transcripts for *SUS* genes in tuber development was negatively correlated with the variations in Suc contents, further supporting the important role of *SUS* in Suc degradation. These data suggest that SUS might be the preferred enzyme for initial Suc metabolism and the intracellular Suc degradation is likely to primarily rely on the activity of SUS with high levels of transcripts in tubers of *C. esculentus*. Our results could reinforce and support the enzyme assays of seeds of rapeseed, wheat, lima bean, and tuber of potato which indicated that SUS predominated over INV in enzyme activity during the storage organ development and is the major enzyme responsible for hexose generation [45–48]. It was demonstrated that SUS plays a physiologically important role as a key enzyme involved in Suc metabolism that appears to be largely in charge of channeling photoassimilated carbon into sink organs and its sucrolysis is the primary entry route of carbon from Suc [49].

**Fig. 7:**
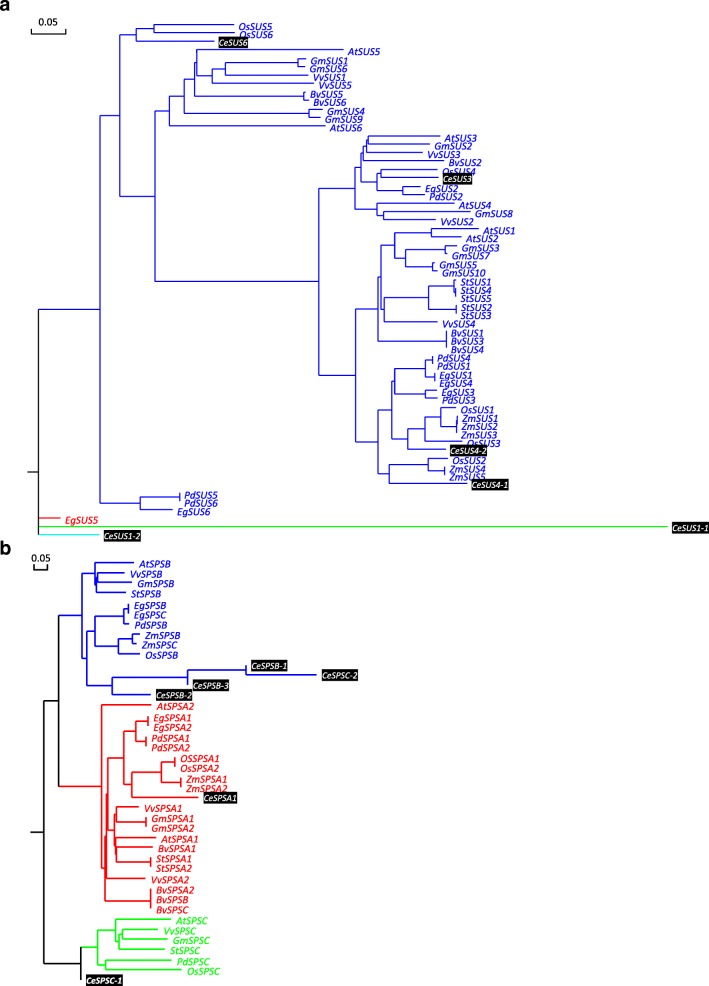
Phylogenetic trees for (**a**) *SUS* and (**b**) *SPS* gene subfamilies among diverse plants. Trees were constructed by using the Maximum Likelihood method based on the Jones-Taylor-Thornton (JTT) matrix-based model. These trees were constructed based on amino acid sequences. Phylogenetic analyses were conducted in DNAMAN8. *At, Arabidopsis thaliana; Gm, Glycine max; St, Solanum tuberosum; Vv,Vitis vinifera; Os, Oryza sativa; Zm, Zea mays; Eg, Elaeis guineensis; Pd, Phoenix dactylifera; Bv, Beta vulgaris; Ce, Cyperus esculentus*

It was shown that *SUS* is encoded by a small multigene family that comprises at least two isoforms in most plant species, with the number of isoforms varying in different plant species. For example, Arabidopsis and rice contain six distinct *SUS* genes [50, 51], while there are three *SUS* genes in maize and pea [52, 53]. In *C. esculentus* tuber, four isoforms of *SUS* orthologs were identified, including one copy of *SUS3* (AT4G02280) and *SUS6* (AT1G73370), two copies of *SUS1* (AT5G20830) and *SUS4* (AT3G43190) (Additional file 1: Table S3). Among these isoforms, the most transcribed one is the *SUS1* ortholog, which is followed by *SUS4* and *SUS3*, while *SUS6* was only faintly expressed (Fig. 5). During the tuber development, the three *SUS1*, *SUS3* and *SUS4* orthologs were all down-regulated and showed a coordinated expression pattern (Fig. 5), implying the important contribution of these *SUS* isoforms to Suc synthase. The relative high transcript levels of *SUS1* in *C. esculentus* tuber were not common to other plant storage tissues. For example, abundantly expressed *SUS1* were only identified in seeds of Arabidopsis and maize, sugarbeet taproot and potato tuber (Fig. 6b). The results of phylogenetic analyses also suggest that the expression pattern of *SUS1* in *C. esculentus* tuber is specific (Fig. 7a).

The existence and relative high abundance of transcripts for the orthologs of *SUS3* and *SUS4* also occurred in other plant seeds, fruits, roots and tubers, implying an evolutionarily conserved role for these two isoforms of SUS enzyme in Suc degradation among diverse plant storage tissues. In contrast to most seed and fruit tissues, *SUS2* ortholog was either not found or were barely detectable in *C. esculentus* tuber, similar to roots of sugar beet and sweet potato as well as potato tuber, suggesting that ortholog of SUS2 is not a prerequisite for Suc synthase in root and tuber tissues, and perhaps SUS2 ortholog in seed and fruit tissues may have evolved to play an additional role in Suc breakdown. The absence and/or very weak expression of *SUS5* and *SUS6* suggested their less contribution to Suc synthase in various sink organs of diverse plants.

Overall, these results strongly indicated that the *SUS* genes in *C. esculentus* tuber displayed specific temporal and spatial expression patterns. It is likely that different isoforms of Suc synthase could have particular functions in the tuber. Previous evidence has shown that the members of Arabidopsis *SUS* genes exhibited partially overlapping but also different expression features. *AtSUS2* was highly and specifically expressed only in the developing seeds; in contrast, *AtSUS1*, *AtSUS5* and *AtSUS6* were more generally expressed in the root, stem, flower, siliques and seed. *AtSUS3* is mainly expressed in the root, flower and seed [49].

### The expression patterns of invertase genes are species-specific in *C. esculentus* tubers

INV is usually categorized into three sub-families based on the subcellular localization, i.e., cell wall INV (CWIN), vacuolar INV (VIN), and cytosolic INV (CIN) which may also be expressed in plastids, mitochondria and nuclei [17, 43, 54].

The three types of INV genes were also detectable in *C. esculentus* tuber, but their transcript expressions were relative low as compared to *SUS*, no more than 120 FPKM even at early tuber development, and decreased to less than 15 FPKM at tuber maturity (Fig. 5). Among these *INV* orthologs, more than 50% of the transcripts were contributed by the *VIN* isoforms, while *CWIN* and *CIN* orthologs were represented by 25 and 22%, respectively (Fig. 6c). The very low expression for *CWIN* in oil tubers might reflect the availability of hexoses for cytosol is primarily through CIN or SUS or both catalytic reactions. This is further supported by the expression patterns of hexose transporters (*HTs*), where *HTs* were only poorly expressed that had less than 15 FPKM of transcripts which were seven-fold on average lower than Suc transporters (*SUTs*) (Additional file 1: Table S3). Suc transport in *C. esculentus* tuber was believed to be similar to potato [4]. When tuberization was initiated, Suc was unloaded symplastically from the phloem through plasmodesmata [55].

The transcript expressions and their contribution of the three subcellular *INVs* in nutsedge were quite different from the counterparts in other plant storage organs, which were varied across the species and tissues (Additional file 2: Figure S1). This is confirmed by the fact that none of the plant storage tissues examined in this study has the same ratio of isoenzymes or transcript patterns as seen in other plants. For example, in underground storage tissues, the highest expression in sweet potato and potato was for the *CIN* orthologs rather than *VIN*, in contrast to nutsedge with the transcripts for *VIN* being the richest. The *CIN2* isoform transcribed most abundantly in potato, but it was absent in sweet potato and nutsedge. Seven isoforms of *CWIN* orthologs (*CWIN1–6*) were detectable in *C. esculentus*, while only *CWIN1* and *CWIN3* orthologs were expressed with low transcripts in potato [56], and no *CWINs* were identified in sweet potato [57].

The multiplicity of genes and the high divergence in expression patterns in the *INV* subfamilies among diverse plants may reflect the evolution of mechanisms underlying the Suc hydrolysis catalyzed by INVs. It was demonstrated that *INV* gene duplication and divergence occurred prior to the separation of the plant species during evolution [58]. This divergence supports the hypothesis that the various *INV* genes have specialized functions [54]. Their expressions were characterized by tissue-, organelle- and development-specific expression patterns [59, 60]. The physiological advantage of having multiple isoenzymes of INVs might be a greater flexibility in the control of Suc metabolism, translocation or storage under different conditions [59].

The differentially regulated expression of *INV* orthologs was also observed in the development of oil tubers of *C. esculentus*. As shown in Fig. 5, the three *INV* subfamilies displayed distinct expression patterns, though they were all down-regulated across the tuber development. *VIN* orthologs had the most abundant transcripts with over 3-fold higher than *CWIN* or *CIN* at early stage of tuber development, but they were hardly expressed at tuber maturity. At tuber mid-stage development, these *INVs* were almost equally transcribed. All these facts reflect that the three *INV* isoforms are developmentally regulated and expressed with overlapping but distinct expression patterns. It might indicate that Suc partitioning via Suc turnover in developing *C. esculentus* tubers could be that SUS would regulate Suc utilization, whereas isoenzymes of INV control Suc storage and sugar composition, as pointed out by previous studies [20, 58, 61, 62]. The plant vacuole is the major site for sugar storage and plays a central role in the temporary or long-term storage of sugars [63]. A positive correlation of VIN activity with hexose accumulation has been found in tomato, grape berry and potato tubers [64–66].

### Cytosolic hexose phosphorylation in *C. esculentus* tubers display distinct and conserved expression patterns

The hexose phosphorylation is related to the synthesis of F6P toward Suc synthesis via three different pathways catalyzed by various enzymes (Fig. 2a). One is the direct conversion of Fru to F6P by the action of FK. The other one is involved in the formation of G6P from Glc catalyzed by HXK and the subsequent conversion of G6P to F6P through the reversible catalytic action of GPI. The third one is through the way that G1P is produced from UDP-Glc and then converted into G6P, which were catalyzed by UGP and PGM, respectively [17, 43].

**Fig. 8:**
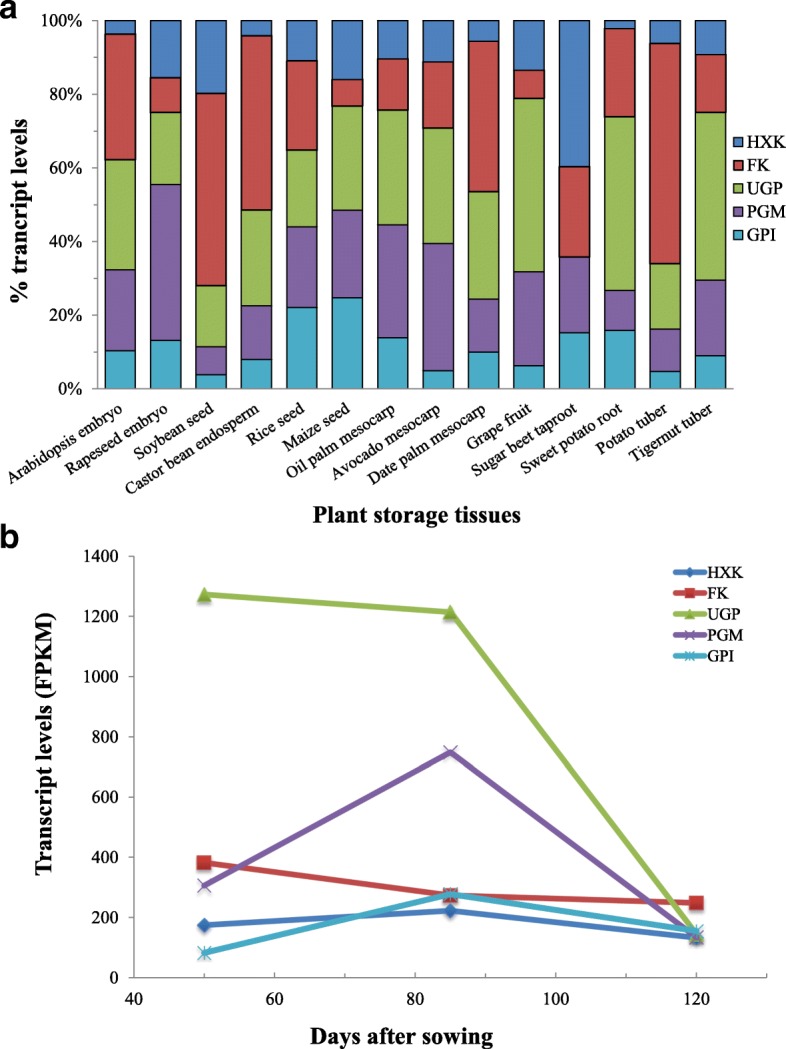
Expression patterns of each hexose phosphorylation genes in diverse plant storage tissues. The relative distribution of transcript levels in different plant storage tissues. **a** The transcript levels (FPKM) are average values and summed for subunits of a protein and for multiple isoforms. **b** Temporal transcriptional levels in developing tuber of *C. esculentus*

*C. esculentus* tuber contained all of the five enzymes necessary for the hexose phosphorylation, as other plant storage tissues (Fig. 8), except for the sugar beet taproot where *UGP* was not detected at gene expression levels [67]. However, the distribution of transcript levels of the orthologs of the five gene family members was varying in different tissues and in diverse species. In *C. esculentus* tuber, the expression level of *UGP* was highest, which was followed by *PGM* and then *FK*, while the *HXK* and *GPI* were the least. Such an expression pattern is quite different from other plants, indicating that the hexose phosphorylation process in different plants is possibly dominated by different enzymes.

In most plant species, each of the five enzymes for hexose phosphate pathway was encoded more than one gene. As shown in Additional file 3: Figure S2, the transcripts for isoforms of the same gene family were contributed in great difference in different plant tissues, showing the dissimilarities of expression patterns of gene isoforms among diverse plant species. Most plants contain two isoforms of *UGP* or *PGM*, whereas in *C. esculentus* tuber only one isoform of *UGP2* or *PGM2* ortholog was identified. In contrast to most plant tissues having single copy of *UGP2*, four copies of *UGP2* ortholog were detected in *C. esculentus* tuber (Additional file 1: Table S3), comparable to the seeds of soybean [68], rice [69], and maize [70] which have three or four *UGP2* isoforms.

A comparison of the transcripts of the orthologs of each gene family in diverse tissues of plants indicated the dissimilarities, while also revealed several conserved features. As in most plant tissues, the higher abundance of transcript level for *FK* orthologs than that of *HXK* was also observed in nutsedge tuber, suggesting that the hexose phosphorylation perhaps relies primarily on Fru substrate. In addition, the more abundance of transcripts for *UGP* and *PGM* orthologs than *HXK*, along with much higher expression levels for *SUS* than *INV* implicated that UGP-mediated G6P synthesis is highly active and UGP might be the major determinant in generation of G6P and play an important role in hexose phosphate pathways.

It was observed that during oil tuber development, the five enzyme genes displayed distinct expression patterns (Fig. 8b), indicating that their expressions were developmentally regulated. Furthermore, the expression patterns were not consistent with the changes in Suc contents, suggesting that these enzymes might not be the major factors associated with Suc accumulation in *C. esculentus* tuber.

### Gene expressions of Suc biosynthetic pathway in oil tubers of *C. esculentus* may be atypical

The production of Suc in the pathway of Suc biosynthesis starting from UDP-Glc in combination with F6P generally involves two enzymes: SPS and SPP (Fig. 2a). SPS catalyzes the formation of S6P from UDP-Glc and F6P, whereas SPP catalyzes the irreversible hydrolysis of S6P to generate Suc. The SPP catalytic reaction is basically irreversible and changes the equilibrium of reversible SPS reaction toward the direction of net Suc synthesis [17].

**Fig. 9:**
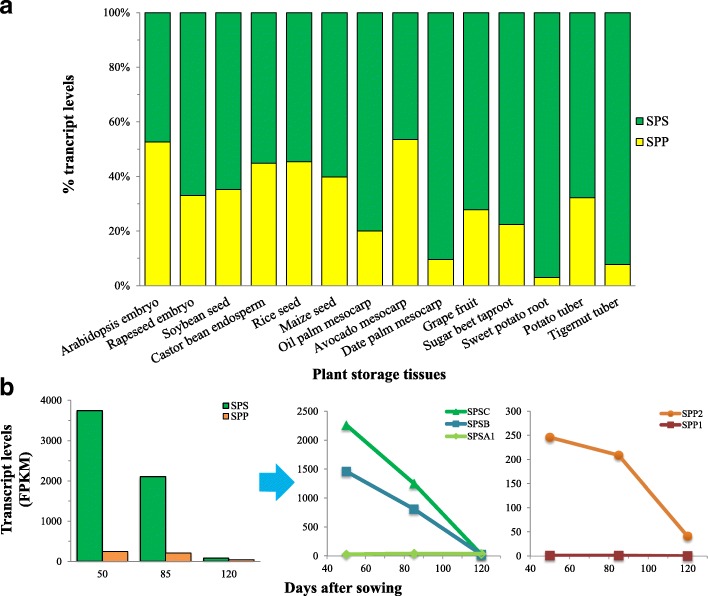
Expression patterns of sucrose phosphate synthase (*SPS*) gene and sucrose phosphate phosphatase (*SPP*) gene. **a** The relative distribution of transcript levels in different plant storage tissues. **b** Temporal transcriptional levels for *SPS* and *SPP* in developing tuber of *C. esculentus*

In nutsedge tuber, the orthologs of Suc biosynthesis genes were represented by an average of 1070 FPKM/protein (Fig. 2b). More than 90% of the transcripts encoding for Suc biosynthesis proteins were attributed by *SPS* orthologs (Fig. 9a). Similar higher expression levels for *SPS*, relative to *SPP*, were also observed in other oil- or carbohydrate-rich plant tissues except for Arabidopsis seed and avocado mesocarp where SPS were expressed slightly less than SPP. However, the highly expressed patterns of *SPS* in *C. esculentus* tuber presented a striking contrast to those of most of other plant tissues in which the *SPS* orthologs were expressed with less five-fold higher as compared to *SPP*, whereas the transcript levels for *SPS* in *C. esculentus* tuber were more ten-fold than that of *SPP*, similar to the case in date palm mesocarp or potato tuber.

The higher expression of *SPS* than *SPP* ortholog in *C. esculentus* tuber also occurred in the course of tuber development (Fig. 9b). Both of *SPS* and *SPP* orthologs were expressed in a down-regulation way. At the early stage, the transcripts for *SPS* were much more abundant, at least 15-fold higher than those for *SPP*. Even at tuber maturity, although the expression levels for *SPS* and *SPP* declined by more forty- and six-fold, respectively, the expression level of *SPS* remained two-fold higher than that of *SPP*.

Overall, these results above may reflect the predominant role of *SPS* ortholog played in Suc synthesis in *C. esculentus* tuber, supporting the fact that SPS is a key regulatory enzyme in the pathway of Suc synthesis and makes a significant contribution to control the flux of carbon into Suc [71, 72], while SPP seems to have little control over carbon flux toward Suc biosynthesis [73].

Evidence has shown that higher plants contain several *SPS* isoforms encoded by a small *SPS* multigene family. In Arabidopsis, *SPS* gene family consists of four member isoforms which are denoted as *SPSA1* (AT5G20280), *SPSA2* (AT5G11110), *SPSB* (AT1G04920) and *SPSC* (AT4G10120) [74–76]. In nutsedge tuber, the *SPSA2* ortholog was not identified (Additional file 4: Figure S3). The transcripts for the ortholog of *SPSC* were the most abundant, followed by *SPSB*. *SPSA1* was least expressed, with only 2% of total transcripts among *SPS* isoforms. The relative low expressions of *SPSA* isoforms in *C. esculentus* tuber may reflect their less contribution to SPS enzyme responsible for Suc synthesis. These results were in stark contrast to the observations made in other plant storage tissues, where *SPSA* were most abundantly expressed while *SPSB* and *SPSC* were poorly transcribed or undetectable (Additional file 4: Figure S3). The difference in *SUS* gene subfamilies between *C. esculentus* and other plants was also suggested by the phylogenetic analyses (Fig. 7b). It has been reported that Arabidopsis *SPSA1* and *SPSA2* were expressed in all organs [75, 77, 78], while *SPSB* was mainly expressed in the reproductive organs, and the *SPSC* gene was preferentially expressed in leaves, indicating that *SPSA*- and *C*-family members were the major *SPS* isoforms expressed in leaves, which seems to be the same thing for Arabidopsis seeds (Additional file 4: Figure S3). It is noted that *SPSA1* was expressed in all of plant storage tissues examined, though to a different extent. Previous phylogenetic analyses revealed that the regulatory phosphorylation sites of SPS are well conserved among SPSA family members from different species [77], suggesting that the SPSA isoforms, in particularly SPSA1, were evolutionary conservation and might play a housekeeping role in plants.

The temporal expression patterns of the three *SPS* genes in *C. esculentus* tubers also revealed that *SPSB*- and *C*-family members constitute the major *SPS* isoforms expressed (Fig. 9b), in which they were all transcribed and appear to be enzymatically active during the tuber development. However, gene expression of *SPSA1* was slightly up-regulated, while the transcripts for the *SPSB* and *SPSC* isoforms showed down-regulation patterns which were in accordance with the dynamic changes in Suc contents. It may reflect that *SPSA1* appear to be functionally distinct, at least in part, from *SPSB*- and *C*-family members in *C. esculentus* tubers and not crucial for Suc synthesis.

The role of SPP in Suc synthesis and whether SPP is or not rate limiting still remain elusive. It was previously demonstrated that SPP seems to make less contribution to the control of Suc biosynthesis [73]. In Arabidopsis, four copies of *SPP* genes, *SPP1* (At1g51420), *SPP2* (At2g35840), *SPP3A* (At3g54270) and *SPP3B* (At3g52340) (Additional file 4: Figure S3), have a similar exon-intron structure [74, 79]. In *C. esculentus* tubers, *SPP* family comprises four genes with one copy of *SPP1* and three copies of *SPP2* orthologs, without *SPP3* members (Additional file 1: Table S3). They displayed distinct expression profiles where the expression levels for *SPP2* isoform were more 100-fold higher than *SPP1*. *SPP1* was expressed very weakly across the tuber development (Fig. 9b). The dominant expression patterns for *SPP2* isoform also occurred in other plant storage tissues (Additional file 4: Figure S3), implicating the evolutionary conservation and a housekeeping role in diverse plants for *SPP2* ortholog. These results reinforce and extend to a recent study revealing that Arabidopsis *SPP2* was expressed most abundantly and showed the highest activity in aerial parts of the plant, whereas *SPP1* isoform was a non-active enzyme gene and mainly expressed in roots [80].

## Discussion

In contrast to common tuber and root crops such as potato and carrot which exclusively accumulate carbohydrates as the major storage components, *C. esculentus* is a currently known only plant that produces tubers containing high amounts of both oil and starch as main storage reserves. Furthermore, *C. esculentus* also accumulate a significant amount of sugars in tubers. Thus, *C. esculentus* is considered a novel model plant suitable for studying carbon partitioning toward the biosynthesis of various storage reserves in underground sink tissues such as tubers and roots [4]. In this study, we have carried out for the first time comprehensive transcriptome analyses of gene expressions related to Suc metabolism, a central reaction for various carbon metabolism, from Suc degradation toward Suc re-synthesis in *C. esculentus* tubers, and compared with other plant oil- or carbohydrate-rich storage tissues including seeds, fruits, roots, and tubers so as to disclose the underlying mechanism of Suc metabolism in oil tuber plants.

Our results showed that gene isoforms involved in conventional Suc metabolic pathways consisting of consecutive reactions of Suc breakdown, hexose phosphorylation and Suc re-biosynthesis are also present in *C. esculentus* tuber and they are functionally conserved among diverse plant species, indicating that plant sink tissues remain a common set of gene isoforms for Suc metabolism during plant evolution. As other plant oil- or carbohydrate-rich sink tissues, the expressions of genes responsible for Suc metabolism in *C. esculentus* tuber appear to be developmentally regulated. The higher levels of transcripts of Suc degradation over hexose phosphorylation in *C. esculentus* tuber are also similar to those of oil- or carbohydrate-rich storage tissues. Similarly, in both *C. esculentus* tuber and other plant storage tissues are shown much higher transcript levels for *SUS* than *INV* isoforms and for *SPS* against *SPP* genes. In addition, multiple temporal expression patterns of genes involved in hexose phosphate pathways also occur in diverse plant storage tissues, suggesting the relative importance of these different enzymes during plant development.

Nevertheless, there are several distinct gene expression patterns that are likely to be tissue or species specific in *C. esculentus* tuber. In contrast to other plant storage organs, *C. esculentus* had high abundance of gene expressions in its tuber for both Suc degradation and synthesis that displayed a coordinated temporal expression pattern in agreement with the changes in Suc levels during tuber development. Therefore, the oil tuber of *C. esculentus* could be used to explore mechanisms modulating the net flux through the Suc pool regulated by the cycle of simultaneous degradation and synthesis of Suc, and whether it is a prerequisite or not to maintain a high net rate of Suc degradation during tuber development. In this respective, given only the orthologs for *SUS1, SUS3* and *SUS4* among *SUS* isoforms were highly expressed in a similar pattern in tubers, this oleaginous tissue is suitable to determine the significance of the simultaneous expression of these three *SUS* isoforms and to examine the possibility of their overlapping function in catalyzing Suc breakdown. In striking contrast to other plant storage organs, the orthologs for *SPSB* and *SPSC* isoforms, where their function in Suc biosynthesis is still not well understood in model plants, were abundantly transcribed with a coordinated expression pattern in *C. esculentus* tuber. Thus, the oil-rich tuber of *C. esculentus* could serve as an ideal system to characterize the specific functions and regulation for these two *SPS* isoforms in Suc biosynthesis. With the poor expression or absence of vacuolar *INVs* in common belowground crops such as roots of sugar beet and sweet potato, as well tuber of potato, it remains elusive as to how individual *VIN* isoforms modulate vacuolar sugar homeostasis, cytosolic hexose levels and cell expansion in underground storage tissues; however, the identification of *VIN2* ortholog with relative abundant transcripts among *VINs* in nutsedge tuber provides with an useful means to study the function and regulation of *VIN2* isoform and the mechanisms underlying VIN-mediated plant development.

## Conclusion

In conclusion, our comprehensive transcriptome analysis of oil tuber of *C. esculentus* in comparison with other plant oil- or carbohydrate-rich storage tissues showed Suc metabolism genes with both similar and different species-specific expression patterns, pointing to the fact that in *C. esculentus*, (i) Suc metabolism also occurs across the tuber development of *C. esculentus* and the transcriptional expressions of the related genes are highly developmentally regulated; (ii) most of Suc metabolism genes are present with multiple isoforms as well, most likely to fulfill their diverse functions in the control of Suc metabolism under different conditions; (3) genes for both Suc degradation and re-synthesis are highly expressed with a coordinated downregulation pattern, dissimilar to the case in other plant storage tissues; and (4) the orthologs of *SUS1, INV2, UGP2, SPSB,* and *SPSC* having relatively abundant transcript levels in *C. esculentus* tuber could be potential gene targets for future metabolic engineering approaches. Together, our study gives insights into the transcriptional control of Suc metabolism in the developing oil tuber tissues. Such knowledge has implications for manipulating potential targets for Suc metabolism in *C. esculentus* through metabolic engineering or molecular breeding to promote this underutilized crop large-scale cultivation and utilization to improve the biomass quantity and quality of *C. esculentus* tuber.

## Methods

### Plant materials

*Cyperus esculentus* L. var. *sativus* were grown in an incubator at 22 ± 0.5°C, 40–60% relative humidity and 16 h light (130 μmol⋅ m^2^⋅ s^− 1^) /8 h dark cycle. Fresh tubers were harvested at different developmental stages and stored in liquid nitrogen immediately until further use.

### Sugar determination

At least 10 tubers were pooled and ground in liquid N_2_ into fine powder that was then homogenized in 80% (*v*/v) ethanol for 5 min. After that, the homogenate was centrifuged at 6000 g for 10 min and the liquid supernatant was used for sugar analysis. Quantification of soluble sugars (Suc, Glc, and Fru) in tubers was determined using anthrone colorimetric method of Morris [81] in combination with the 3, 5-dinitro salicylate method of Miller [82].

### Total RNA extraction

Total RNA was isolated from the developing tubers using a modified cetyltrimethylammonium bromide (CTAB)-based method as described in detail in [40]. Pure RNA sample was dissolved in RNase-free water and stored in liquid nitrogen.

### RNA deep sequencing

The quality and yield of total RNAs were verified using a NanoDrop 2000 spectrophotometer and an Agilent 2100 Bioanalyzer. RNA sequencing was performed using the Illumina Hiseq4000 sequencing platform (Shanghai Major Biomedicine Technology Co. Ltd., China). RNA-seq data are available on the National Center for Biotechnology Information (NCBI) Short Read Archive Project- PRJNA320781, PRJNA320787 and PRJNA312713. Annotation for De novo assembly sequences (> 200 bp) was conducted using a BLAST homology search against the NCBI protein database (ftp://ftp.ncbi.nlm.nih.gov/blast/db/), Swiss-Prot (http://www.ebi.ac.uk/uniprot/), COG (http://www.ncbi.nlm.nih.gov/COG/), STRING (http://string-db.org/), GO (http://www.geneontology.org/) and KEGG (http://www.genome.jp/kegg/). The levels of gene expressions were quantified using fragments per kilobase of exon model per million mapped reads (FPKM) [83].

### Quantitative real-time PCR (qRT-PCR) analysis

qRT-PCR analysis was carried out with SYBR Green Realtime PCR Master Mix (Toyobo Co. Ltd.) using the system of Eppendorf Mastercycler ep realplex (Eppendorf Company, Germany), as the method in our previous work [40]. Relative mRNA was quantified by the ΔΔC_T_ method [84]. 18S rRNA was used as the internal control. Primer pairs for qRT-PCR analysis were listed in Additional file 1: Table S4.

## Additional files

Additional file 1: **Table S1.** Summary of *C. esculentus* transcriptome datasets, assembly and annotation, and number of transcripts and unigenes related to carbohydrate metabolism. **Table S2.** Annotation of *C. esculentus* transcriptome. **Table S3.** Annotation and expression levels for selected genes associated with sucrose metabolism. **Table S4.** Primer pairs used for qRT-PCR. **Table S5.** The assembled sequences for selected genes associated with sucrose metabolism. (XLSX 6684 kb)

Additional file 2: **Figure S1.** Relative gene expression levels for each invertase isoform in diverse plant storage tissues. (PDF 86 kb)

Additional file 3: **Figure S2.** Relative gene expression levels for each isoform of hexose phosphorylation in diverse plant storage tissues. (PDF 63 kb)

Additional file 4: **Figure S3.** Relative gene expression levels for each isoform of sucrose synthesis pathways in diverse plant storage tissues. (PDF 61 kb)

## Abbreviations

CIN: cytoplasmic invertase
CWIN: cell wall invertase
F6P: fructose-6-phosphate
FK: fructokinase
Fru: fructose
G1P: glucose-1-phosphate
G6P: glucose-6-phosphate
Glc: glucose
Hexose-P: hexose-phosphate
HT: hexose transporter
HXK: hexokinase
PD: plasmodesmata
PGI: phosphoglucoisomerase
PGM: phosphoglucomutase
S6P: succose-6-phosphate
SPP: Suc-phosphatase
SPS: Suc-phosphate synthase
Suc: Suc
SUS: Suc synthase
SUT: Suc transporter
SWEET: Sugars Will Eventually be Exported Transporter (hexose and Suc transporter)
T6P: trehalose-6-phosphate
TPS: trehalose-6-phosphate synthase
TST: tonoplast Suc transporter
UDP-Glc: uridine diphosphate glucose
UGP: UDP-Glc pyrophosphorylase
VIN: vacuolar invertase
