# Supplementary material for: Sucrose metabolism in developing oil-rich tubers of Cyperus esculentus: comparative transcriptome analysis
Source: BMC Plant Biol. 2018 Jul 24;18:151. doi: 10.1186/s12870-018-1363-9 (PMC6056992; doi:10.1186/s12870-018-1363-9)

Subcellular Localization	Arabidopsis homolog (Locus ID)	Protein/gene Abbreviation	Relative gene expression levels for hexose phosphorylation isoforms													
			At	*Bn*	*Gm*	*Rc*	*Os*	*Zm*	*Eg*	*Pa*	*Pd*	*Vv*	*Bv*	*Ib*	*St*	*Ce*
Cytosol	AT4G29130	HXK1														
	AT2G19860	HXK2														
Cytosol	AT2G31390	FK1														
	AT1G06030	FK2														
	AT1G06020	FK3														
	AT3G59480	FK4														
	AT4G10260	FK5														
	AT5G51830	FK7														
Cytosol	AT3G03250	UGP1														
	AT5G17310	UGP2														
Cytosol	AT1G23190	PGM1														
	AT1G70730	PGM2														
Cytosol	AT5G42740	GPI														

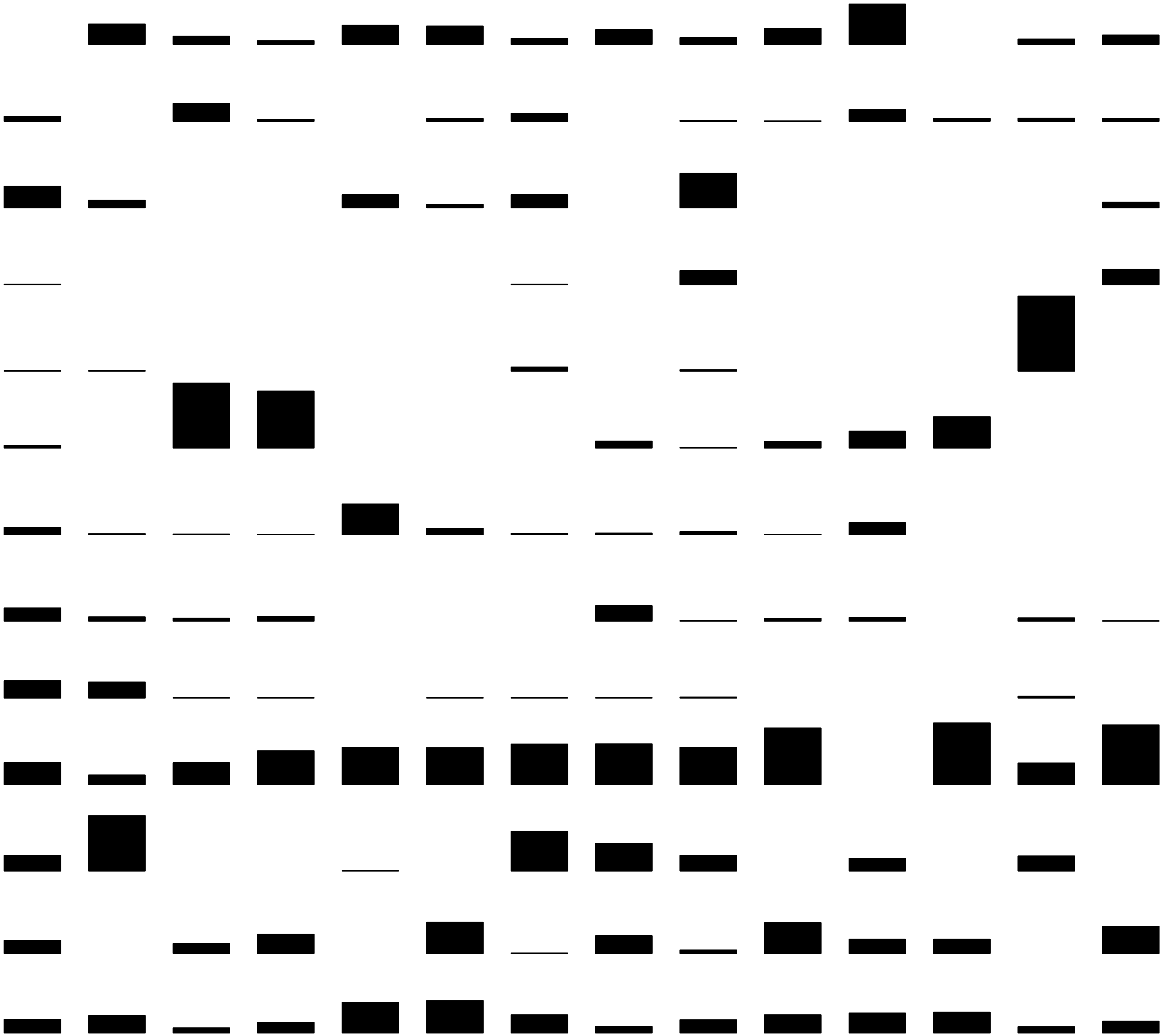

Supplement: Supplementary file 3 — Figure S2. Relative gene expression levels for each isoform of hexose phosphorylation in diverse plant storage tissues. (PDF 63 kb) [file 12870_2018_1363_MOESM3_ESM.pdf]
