# Supplementary material for: Sucrose metabolism in developing oil-rich tubers of Cyperus esculentus: comparative transcriptome analysis
Source: BMC Plant Biol. 2018 Jul 24;18:151. doi: 10.1186/s12870-018-1363-9 (PMC6056992; doi:10.1186/s12870-018-1363-9)
Supplement: Supplementary file 2 — Figure S1. Relative gene expression levels for each invertase isoform in diverse plant storage tissues. (PDF 86 kb) [file 12870_2018_1363_MOESM2_ESM.pdf]

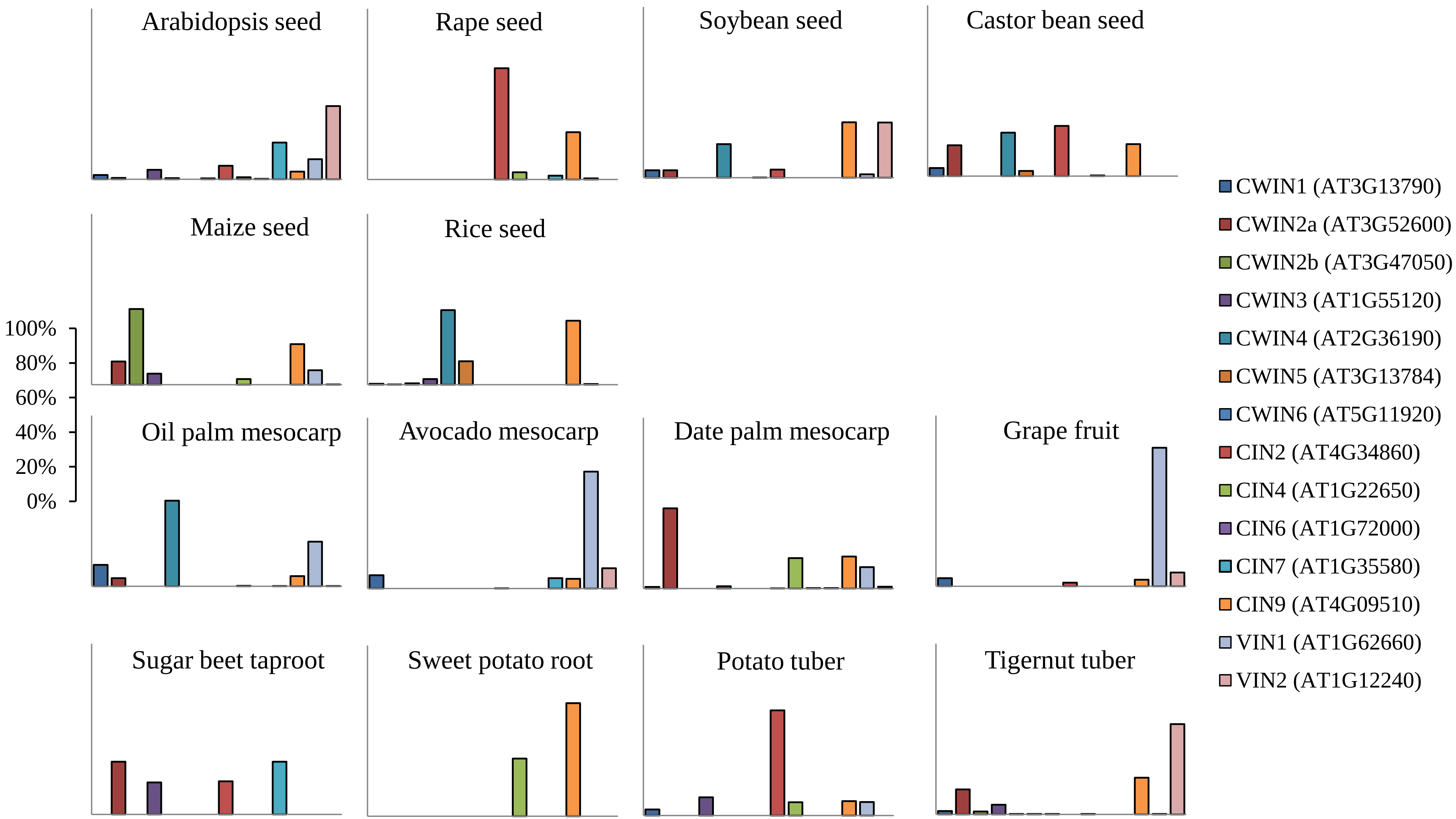
Arabidopsis seed
Rape seed
Soybean seed
Castor bean seed
Maize seed
Rice seed
100%
80%
60%
40%
20%
0%
Oil palm mesocarp
Avocado mesocarp
Date palm mesocarp
Grape fruit
Sugar beet taproot
Sweet potato root
Potato tuber
Tigernut tuber
CWIN1 (AT3G13790)
CWIN2a (AT3G52600)
CWIN2b (AT3G47050)
CWIN3 (AT1G55120)
CWIN4 (AT2G36190)
CWIN5 (AT3G13784)
CWIN6 (AT5G11920)
CIN2 (AT4G34860)
CIN4 (AT1G22650)
CIN6 (AT1G72000)
CIN7 (AT1G35580)
CIN9 (AT4G09510)
VIN1 (AT1G62660)
VIN2 (AT1G12240)
